# Generation of single-cell and single-nuclei suspensions from embryonic and adult mouse brains

**DOI:** 10.1016/j.xpro.2022.101944

**Published:** 2022-12-14

**Authors:** Dongjin R. Lee, Yajun Zhang, Christopher T. Rhodes, Timothy J. Petros

**Affiliations:** 1 Unit on Cellular and Molecular Neurodevelopment, *Eunice Kennedy Shriver* National Institute for Child Health and Human Development (NICHD), National Institutes of Health (NIH), Bethesda, MD 20892, USA

**Keywords:** Single Cell, Flow Cytometry/Mass Cytometry, Developmental biology, Sequencing, RNAseq, Neuroscience, Cell Differentiation

## Abstract

Efficient protocols to generate single-cell and single-nuclei suspensions are critical for the burgeoning field of single-cell/single-nuclei sequencing. Here we describe procedures to generate single-cell and single-nuclei suspensions from embryonic and adult mouse brains. This protocol can be modified for any brain region and/or neural cell type.

For complete details on the use and execution of this protocol, please refer to Lee et al. (2022),^1^ Rhodes et al. (2022),^2^ Mahadevan et al. (2021),^3^ Ekins et al. (2020),^4^ and Wester et al. (2019).^5^

## Highlights

• Obtain single-cell or single-nuclei suspensions from embryonic or adult mouse brains
• Utilizes sorting to purify samples and select fluorescent cells/nuclei if needed
• Describes different cell preparation strategies depending on downstream applications
• Protocol is applicable to different ages and different brain regions

## Abstract (editor highlights)

Publisher’s note: Undertaking any experimental protocol requires adherence to local institutional guidelines for laboratory safety and ethics.

## Graphical abstract

**Figure:**
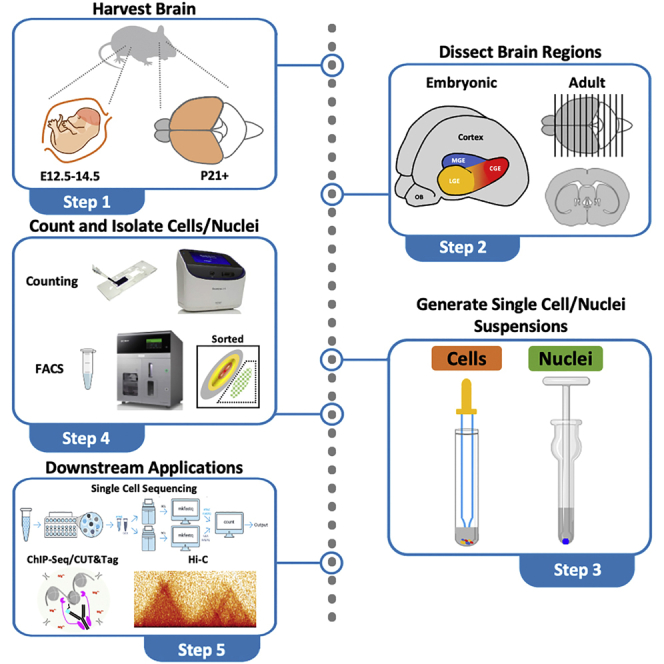

## Before you begin

The advent of single-cell sequencing technologies over the last decade^6^^,^^7^ requires efficient and reproducible protocols to dissociate tissue and generate clean single-cell and single-nuclei suspensions. This is particularly relevant for the brain, which shows high cellular heterogeneity^8^ and presents significant challenges due to the complex neuronal shapes and the excess debris from myelin and sheared neurites.

In this protocol, we describe procedures to generate single-cell or single-nuclei suspensions from both embryonic and juvenile/adult mouse brains.^1^^,^^2^^,^^3^^,^^4^^,^^5^ While our focus is the forebrain, we performed this procedure in the spinal cord as well.^9^ We provide additional detail to prepare the samples for sequencing on the 10x Genomics platform, specifically with the single-cell/nuclei RNA-seq, single-nuclei ATAC-seq (chromatin accessibility) and single-nuclei Multiome (RNA-seq and chromatin accessibility) kits. Notably, these single-cell and single-nuclei preparations can be used for a variety of downstream applications such as ChIP-seq, CUT&Tag and chromatin confirmation technologies.^2^ These preparations may also be useful for a number of recently developed single-cell multimodal assays that are compatible with the 10x Genomics platform such as Perturb-seq,^10^^,^^11^ CITE-seq,^12^ ASAP-seq^13^ and scCUT&Tag-pro,^14^ although we have not tested these protocols on these specific assays. Fluorescence-activated cell sorting (FACS) can (and in many cases should) be performed to harvest fluorescent cells or nuclei from suspensions to enrich for specific neuronal subtypes and/or eliminate debris. We *strongly recommend* sorting all cell and nuclei suspensions from adult brain dissections to remove debris and generate clean cell or nuclei suspensions for downstream applications. All our FACS was performed on a Sony SH800 cell sorter, but other comparable cell sorters should produce similar results.

While these protocols have only been tested on embryonic and adult (P21-P60) mouse brains, we are confident that these procedures are also applicable to any age of postnatal development, and for any nervous system region of interest. We recommend researchers perform trial runs on their desired tissue or cell-types before experimentation because distinct brain regions contain different proportions of cells and myelin, which may affect the amount of starting material required and the efficiency of the cleanup procedure.

For anyone using these protocols for single-cell sequencing using the 10x Genomics platform, we recommend exploring the 10x Genomics Support Documentation resources as they regularly provide updated protocols for tissue processing with their single-cell sequencing platforms. Many of our nuclei preparation solutions are in part based on these 10x Genomics protocols. One notable difference is that we utilize mechanical dissociation via Dounce homogenizers to lyse brain tissue whereas most current 10x Genomics protocols digest tissue with detergents on ice followed by trituration. In our experience, we obtain better nuclei recovery and higher quality cDNA libraries and sequencing results using the mechanical dissociation in lysis solution to generate nuclei suspensions from both embryonic and adult brain samples.

### Institutional permissions

All experimental procedures were conducted in accordance with the National Institutes of Health guidelines and were approved by the NICHD Animal Care and Use Committee. Both male and female embryonic and adult mice were used without bias for all experiments. Housing conditions: 12/12 h light/dark cycle, humidity between 30%–50%, temperature 20°C–22°C. All researchers will need to acquire permission from their institution to perform these experiments on mice.

### Preparation of equipment and solutions

**Timing: 30–60 min**

1. All dissection tools and Dounce homogenizers should be autoclaved or sterilized using acceptable practices, and proper PPE should be worn throughout the protocol.
2. Prepare isoflurane chamber setup and/or load 0.05 mL Euthasol per mouse into syringe(s) for anesthesia.
3. If generating single-cell suspensions, use a Bunsen burner to fire polish the tips of Pasteur pipettes, creating some with large bore openings (∼600 μm, opening should be clearly visible) and some with small bore openings (∼300 μm, opening should be barely visible). Test the flow speed by pipetting water up and down through fire polished Pasteur pipette.
4. Prepare the following solutions fresh on the day of experiment (depending on the desired procedure) and keep all solutions on ice throughout the procedure:
  a. Embryonic brain dissections: Prepare the artificial cerebral spinal fluid (ACSF) solution and carbogenate (bubble) with 5%:95% CO_2_:O_2_. If generating single-cell suspensions, be prepared to make the Pronase Solution and Cell Reconstitution Solution after brain dissection.
  b. Adult brain dissections: Prepare NMDG High-Mg^2+^ Solution and NMDG Activity-blocking Solution, place on ice and keep carbogenated with 5%:95% CO_2_:O_2_. If sectioning brains, immerse brain matrix in ice or prepare vibratome for sectioning. If generating single-cell suspensions, prepare solutions from the Papain Dissociation Kit per manufacturer’s instructions.
  c. Single-nuclei suspensions.
    i. For downstream applications analyzing mRNA only (e.g., RNA-seq): Prepare RNA-only Nuclei Wash Buffer, RNA-only Nuclei Lysis Buffer and RNA-only Nuclei Resuspension Buffer.
    ii. For downstream applications analyzing DNA only (e.g., ATAC-seq, CUT&Tag, ChIP-seq, etc.): Prepare DNA-only Nuclei Wash Buffer, DNA-only Nuclei Lysis Buffer. If performing 10x Genomics ATAC-seq, also prepare the DNA-only Diluted Nuclei Buffer.
    iii. For downstream applications analyzing DNA and mRNA (e.g., Multiome): Prepare the Multiome Nuclei Wash Buffer and Multiome Nuclei Lysis Buffer. If performing 10x Genomics Multiome, also prepare the Multiome Diluted Nuclei Buffer.

***Note:*** While we have not published on the Multiome technique, we have generated high quality data with the 10x Genomics Multiome kit using the solutions and protocol described below.

## Key resources table

**Table:** 

REAGENT or RESOURCE	SOURCE	IDENTIFIER
**Experimental models: Organisms/strains**		

Mouse: C57BL/6	Jackson Laboratory	#000664

**Chemicals, peptides, and recombinant proteins**		

Forane® Isoflurane	Baxter	#10019-360-40
Euthasol (sodium pentobarbital)	Vet One	#501016
Pronase	Sigma-Aldrich	#10165921001
Fetal bovine serum (FBS)	Thermo Fisher Scientific	#10437028
MACS BSA stock solution (10%)	Miltenyi Biotec	#130-091-376
DNase I (2,000 U/mL)	Worthington	#LS006344
DAPI	Thermo Fisher Scientific	#62248
DRAQ5	Thermo Fisher Scientific	#62251
Phosphate buffered saline (PBS) (pH 7.4)	Gibco	#10010-023
D-Sucrose	Fisher Bioreagents	#BP220-1
RNase-free water	Invitrogen	#10977-015
NaCl	Fisher Bioreagents	#BP358-212
NaHCO_3_	Thermo Fisher Scientific	#447102500
KCl	Fisher Bioreagents	#BP366-1
NaH_2_PO_4_ ∗ H_2_O	Fisher Bioreagents	#S369-500
CaCl_2_ (2 M)	Quality Biological	#351-130-721
MgCl_2_ (1 M)	Quality Biological	#351-033-721
Glucose/dextrose anhydrous	Fisher Bioreagents	#D14-500
Tris-HCl	Crystalgen	#221-228
Tween-20	Sigma-Aldrich	#P7949
IGEPAL CA-630	Sigma-Aldrich	#I8896
*N*-methyl-D-glucamine (NMDG)	Sigma-Aldrich	#66930
HEPES	Sigma-Aldrich	#H4034
Sodium ascorbate	Sigma-Aldrich	#A4034
Sodium pyruvate	Sigma-Aldrich	#P5280
MgSO_4_·7H_2_O	Sigma-Aldrich	#M2773
Thiourea	Sigma-Aldrich	#T8656
CaCl_2_·2H_2_O	Sigma-Aldrich	#223506
Tetrodotoxin	MilliporeSigma	#554412
DL-2-Amino-5-phosphonopentanoic acid (AP5)	Sigma-Aldrich	#A5282
Actinomycin-D	Sigma-Aldrich	#A1410
Magnesium acetate tetrahydrate	Sigma-Aldrich	#M5661
Ethylenediaminetetraacetic acid (EDTA) (0.5 M)	Corning	#46-034-CI
DL-Dithiothreitol (DTT) solution (1 M)	Sigma-Aldrich	#646563
Protector RNase inhibitor (40 U/μL)	MilliporeSigma	#3335402001

**Critical commercial assays**		

Papain Dissociation System	Worthington	#LK003150
Nuclei Pure Prep Isolation Kit (Sucrose Cushion Solution)	Sigma	#NUC-201

**Other**		

Dumont #1 forceps	Roboz Surgical Instrument	#RS-4960
Dumont #2 forceps	Roboz Surgical Instrument	#RS-4962
Dumont #5 forceps	Roboz Surgical Instrument	#RS-4978
Curved micro forceps	Roboz Surgical Instrument	#RS-5101
0.5-mm steel mouse brain coronal matrix	Alto Matrices	#SA-2165
Double-edge stainless razor blades	Electron Microscopy Science	#72000
Falcon 5-mL tube w/ 35-μm cell strainer	Corning	#352235
Falcon 50-mL conical centrifuge tube	Corning	#352098
9″ Pasteur Pipettes	Fisher Scientific	#13-678-20C
60 mm Petri Dish	Fisher Scientific	#11844335
Sylgard-coated Petri dish	Living Systems Instrumentation	#DD-ECON-90
Cell strainer, 40 μm	Fisher Scientific	#22-363-547
Dounce homogenizer, 2 mL	Kimble	#885300-0002
Dounce homogenizer, 1 mL	Kimble	#885300-0001
LoBind tube, 2 mL	Eppendorf	#0030108078
LoBind tube, 1.5 mL	Eppendorf	#0030108051
Super Glue	Krazy	#KG48348MR
Vibratome	Leica	#VT1000S

***Alternatives:*** Many reagents in the ‘Other’ and ‘Chemicals, peptides and recombinant proteins’ categories can be found from other vendors than then ones listed above.

## Materials and equipment

### Solutions for embryonic brain dissections

**Table:** Artificial Cerebrospinal Fluid (ACSF)

Reagent	Final concentration	Amount
NaCl	87 mM	2.54 g
NaHCO_3_	26 mM	1.09 g
KCl	2.5 mM	0.09 g
NaH_2_PO_4_	1.25 mM	0.09 g
Glucose (Dextrose Anhydrous)	10 mM	0.90 g
Sucrose	75 mM	12.84 g
CaCl_2_	0.5 mM	125 μL (2 M stock)
MgCl_2_	7 mM	3.5 mL (1 M stock)
5% CO_2_ & 95% O_2_ (carbogenated)	N/A	N/A
RNase-free water	N/A	Adjust to 500 mL
**Total**	N/A	500 mL

**CRITICAL:** Add CaCl_2_ and MgCl_2_ after bubbling with carbogen to avoid precipitation of these reagents.

***Note:*** pH must be adjusted to 7.4 and solution should always be kept on ice. Final volume of 500 mL can be adjusted depending on volume needed.

### Solutions for embryonic brain dissections – Single-cell suspensions

**Table:** Pronase Solution

Reagent	Final concentration	Amount
ACSF	N/A	10 mL
Pronase	1 mg/mL	10 mg
**Total**	N/A	10 mL

**CRITICAL:** Pronase is classified as Eye Irritation 2A, Skin Irritation 2, and STOT-SE category 3. Keep all hazardous materials stored properly and handle according to approved procedures.

***Note:*** Make fresh just prior to use, after dissecting out relevant brain regions.

**Table:** Cell Reconstitution Solution

Reagent	Final concentration	Amount
ACSF	N/A	9.9 mL
FBS	1%	100 μL
DNase I	2 U/mL	1 μL
**Total**	N/A	10 mL

***Note:*** Make fresh just prior to use when tissue is incubating in Pronase Solution.

**CRITICAL:** If generating single-cell suspensions for analyzing DNA (e.g., Hi-C), do NOT include DNase I in the Cell Reconstitution Solution as this could disrupt downstream DNA assays.

### Solutions for adult brain dissections

**Table:** NMDG High-Mg^2+^ Solution

Reagent	Final concentration	Amount
NMDG	93 mM	9.08 g
KCl	2.5 mM	0.09 g
NaH_2_PO_4_	1.2 mM	0.08 g
NaHCO_3_	30 mM	1.26 g
HEPES	20 mM	2.38 g
Glucose	25 mM	2.25 g
Sodium Ascorbate	5 mM	0.50 g
Sodium Pyruvate	3 mM	0.17 g
MgSO4·7H2O	10 mM	25 mL (2 M stock)
Thiourea	2 mM	0.08 g
CaCl2·2H2O	0.5 mM	125 μL
5% CO_2_ & 95% O_2_ (carbogenated)	N/A	N/A
RNase-free water	N/A	Adjust to 500 mL
**Total**	N/A	500 mL

**CRITICAL:** Thiourea is classified as Acute Toxicity 4 Oral, Aquatic Chronic 2, Category 2 Carcinogen, and Category 2 Reprotoxin. Keep all hazardous materials stored properly and handle according to approved procedures.

**CRITICAL:** Add CaCl_2_ after bubbling with carbogen to avoid precipitation.

***Note:*** Using 10 mM MgSO4·7H2O blocks NMDA channels to prevent excitotoxicity.

***Note:*** pH must be adjusted to 7.4 and solution should always be kept on ice. Final volume of 500 mL can be adjusted depending on volume needed.

***Optional:*** Adjust osmolarity to 300–310 mOsm.

**Table:** NMDG Activity-blocking Solution

Reagent	Final concentration	Amount
NMDG High-Mg^2+^ Solution	N/A	49.9 mL
Tetrodotoxin (1 mM)	0.5 μM	25 μL
AP5 (50 mM)	50 μM	50 μL
Actinomycin-D (10 mM)	10 μM	50 μL
**Total**	N/A	50 mL

**CRITICAL:** Tetrodotoxin is classified as Acute Toxicity 1 Oral. Keep all hazardous materials stored properly and handle according to approved procedures.

**CRITICAL:** AP5 is classified as Eye Irritation 2, Skin Irritation 2, and STOT-SE category 3. Keep all hazardous materials stored properly and handle according to approved procedures.

**CRITICAL:** Actinomycin-D is classified as Acute Toxicity 2 Oral, Category 1B Carcinogen, and Category 1B Reprotoxin. Keep all hazardous materials stored properly and handle according to approved procedures.

***Note:*** pH must be adjusted to 7.4 and solution should always be kept on ice. Final volume of 50 mL can be adjusted depending on volume needed.

***Optional:*** Adjust osmolarity to 300–310 mOsm.

### Solutions for single-nuclei suspensions – mRNA-only analysis (e.g., RNA-seq)

***Note:*** For all solutions below, always keep on ice. Final volumes should be adjusted depending on number of samples.

**Table:** RNA-only Nuclei Wash Buffer

Reagent	Final concentration	Amount
Sucrose	320 mM	547.68 mg
HEPES (pH 8.0)	10 mM	11.92 mg
CaCl_2_	5 mM	12.5 μL
Magnesium-acetate	3 mM	3.22 mg
EDTA	0.1 mM	1 μL
DTT	1 mM	5 μL
RNase-free water	N/A	Adjust to 5 mL (per sample)
**Total**	N/A	5 mL (per sample)

**CRITICAL:** DTT Solution is classified as Eye Damage 1 and Skin Irritation 1. Keep all hazardous materials stored properly and handle according to approved procedures.

**Table:** RNA-only Nuclei Lysis Buffer

Reagent	Final concentration	Amount
RNA-only Nuclei Wash Buffer	N/A	990 μL
IGEPAL CA-630	0.1%	10 μL
**Total**	N/A	1 mL (per sample)

**CRITICAL:** IGEPAL CA-630 is classified as Acute Toxicity 4 Oral, Aquatic Acute 1, Aquatic Chronic 1, Eye Damage 1 and Skin Irritation 2. Keep all hazardous materials stored properly and handle according to approved procedures.

**Table:** RNA-only Nuclei Resuspension Buffer

Reagent	Final concentration	Amount
BSA (10% stock)	1%	100 μL
RNase Inhibitor (40 U/μL)	0.2 U/μL	5 μL
PBS	N/A	895 μL
**Total**	N/A	1 mL (per sample)

### Solutions for single-nuclei suspensions – DNA-only analysis (e.g., ATAC-seq, ChIP-seq, CUT&Tag, etc.)

***Note:*** For all solutions below, always keep on ice. Final volumes should be adjusted depending on number of samples.

**Table:** DNA-only Nuclei Wash Buffer

Reagent	Final concentration	Amount
Tris-HCl (pH 7.4) (1 M)	10 mM	50 μL
NaCl (5 M)	10 mM	10 μL
MgCl_2_ (1 M)	3 mM	15 μL
Tween-20 (10%)	0.1%	50 μL
BSA (10%)	1.5%	750 μL
Nuclease-free water	N/A	Adjust to 5 mL (per sample)
**Total**	N/A	5 mL (per sample)

**Table:** DNA-only Nuclei Lysis Buffer

Reagent	Final concentration	Amount
DNA-only Nuclei Wash Buffer	N/A	990 μL
IGEPAL CA-630 (10% stock)	0.1%	10 μL
**Total**	N/A	1 mL (per sample)

**CRITICAL:** IGEPAL CA-630 is classified as Acute Toxicity 4 Oral, Aquatic Acute 1, Aquatic Chronic 1, Eye Damage 1 and Skin Irritation 2. Keep all hazardous materials stored properly and handle according to approved procedures.

**Table:** DNA-only Diluted Nuclei Buffer (only if using 10x Genomics ATAC-seq kit)

Reagent	Final concentration	Amount
Nuclei Buffer (20×, from kit)	1×	50 μL
Nuclease-free water	N/A	950 μL
**Total**	N/A	1 mL (per sample)

### Solutions for single-nuclei suspensions – DNA and mRNA analysis (e.g., Multiome)

**Table:** Multiome Nuclei Wash Buffer

Reagent	Final concentration	Amount
DNA-only Nuclei Wash Buffer	N/A	4.87 mL
DTT (1 M)	1 mM	5 μL
RNase inhibitor (40 U/μL)	1 U/μL	125 μL
**Total**	N/A	5 mL (per sample)

**CRITICAL:** DTT Solution is classified as Eye Damage 1 and Skin Irritation 1. Keep all hazardous materials stored properly and handle according to approved procedures.

**CRITICAL:** Use full amount of RNase inhibitor. Using less than indicated concentration will likely result in degraded mRNA quantity and quality.

**Table:** Multiome Nuclei Lysis Buffer

Reagent	Final concentration	Amount
Multiome Nuclei Wash Buffer	N/A	990 μL
IGEPAL CA-630 (10% stock)	0.1%	10 μL
**Total**	N/A	1 mL (per sample)

**CRITICAL:** IGEPAL CA-630 is classified as Acute Toxicity 4 Oral, Aquatic Acute 1, Aquatic Chronic 1, Eye Damage 1 and Skin Irritation 2. Keep all hazardous materials stored properly and handle according to approved procedures.

***Note:*** 10x Genomics has 2 relevant protocols for their Multiome kit, one general for all tissue (CG000365, Rev C) and one specific for embryonic mouse brains (CG000366, Rev D). The only difference between these two protocols is the Lysis buffer, with the general protocol using 1× lysis buffer and the embryonic mouse brain protocol using 0.1× Lysis buffer. We have only performed Multiome experiments on embryonic mouse brains, and thus we have only used the 0.1× Lysis buffer (per manufacturer’s instructions). Here, we present the 1× Lysis buffer solution from the general Multiome protocol since it is more broadly applicable. We recommend researchers compare the 1× and 0.1× lysis buffer concentrations on their preparations per the manufacturer’s instructions.

**Table:** Multiome Diluted Nuclei Buffer (only if using 10x Genomics Multiome Kit)

Reagent	Final concentration	Amount
Nuclei Buffer (20×, from kit)	1×	50 μL
DTT (1 M)	1 mM	1 μL
RNase inhibitor (40 U/μL)	1 U/μL	25 μL
Nuclease-free water	N/A	924 μL
**Total**	N/A	1 mL (per sample)

**CRITICAL:** DTT Solution is classified as Eye Damage 1 and Skin Irritation 1. Keep all hazardous materials stored properly and handle according to approved procedures.

**CRITICAL:** Use full amount of RNase inhibitor. Using less than indicated concentration will likely result in degraded mRNA quantity and quality. 10x Genomics recommends the Protector RNase inhibitor (see key resources table above), as other RNase inhibitors can result in a significant reduction of cDNA yield (see 10x Genomics website).

## Step-by-step method details

We describe multiple strategies to generate single-cell or single-nuclei suspensions from either embryonic or juvenile/adult mouse brain tissue. If harvesting tissue from embryonic mouse brains: steps #1–21 describe the procedure to harvest specific regions of the embryonic brain, steps #32–37 describe generation of single-cell suspensions, and steps #46–57 describe generation of single-nuclei suspensions. If harvesting tissue from juvenile/adult mouse brain: steps #22–31 describe the procedure to harvest specific of the adult brain via either brain slices (#22–26) or whole-mount (#27–31), steps #38–45 describe generation of single-cell suspensions, and steps #46–61 describe generation of single-nuclei suspensions. If proceeding with single-cell sequencing reactions using the 10x Genomics platform: steps #62–63 describe how to prepare cell or nuclei suspensions for RNA-seq, ATAC-seq or Multiome analysis.

### Harvesting embryonic mouse brains

**Timing: 15 min**

Embryos are removed from the dam and brains are collected in ACSF.

1. Anesthetize timed pregnant dams with either isoflurane (4% for induction) or Euthasol (270 mg/kg, 50 μL/30 g mouse, intraperitoneal (IP) injection).
2. After confirming no response to painful toe pinch stimuli, perform cervical dislocation and place dam on petri dish.
3. Use sterilized scissors to cut an incision along the midline of lower abdominal to expose the uterus and embryos.
4. Cut through connective tissue under the embryonic chain to extract the uterus with embryos and transfer to carbogenated ACSF in Petri dish on ice.
5. Using Dumont #2 forceps, remove each embryo from the uterus and amniotic sac and transfer embryos to a new Petri dish with carbogenated ACSF on ice.
6. Decapitate each embryo and transfer heads to a new Petri dish with carbogenated ACSF on ice.
7. Select 1 head and under a dissecting microscope, use Dumont #5 forceps and/or curved micro forceps to remove the skin and skull and expose the brain using 1 of 2 strategies:
  a. With the head dorsal side up, stabilize the head of embryo by holding the nasal and Maxillary region with forceps.
    i. Using fine forceps in other hand, peel away skin to expose the skull.
    ii. Pierce the skull with forceps and peel away skull to expose the brain (Figure 1A).

**Figure 1:**
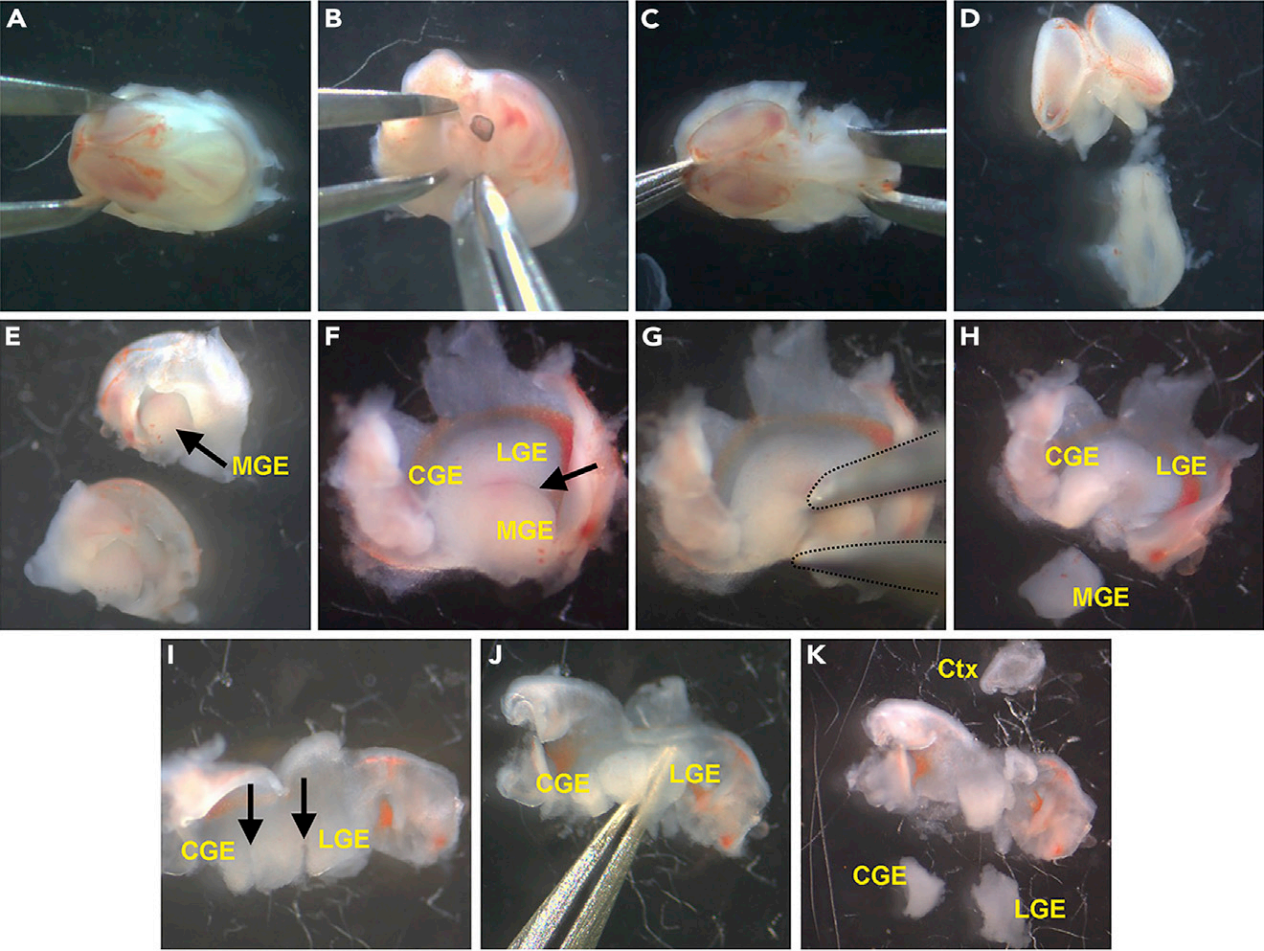
Embryonic mouse brain dissection (A and B) Two different dissection strategies to remove skin and skull. (C) Brain exposed. (D) Remove midbrain and hindbrain. (E) MGE should be visible in lateral ventricles after hemisecting the brain. (F) MGE, LGE and CGE exposed after peeling back cortex, with MGE-LGE sulcus being clearly visible (black arrow). (G and H) Separating MGE by inserting forceps (dotted lines) through MGE-LGE sulcus. (I) Two incisions (black arrows) to separate the CGE and LGE. (J) Forceps used to pinch off LGE from surrounding brain tissue. (K) Removal of LGE, CGE and cortex (Ctx).
  b. With head lying on its side, use forceps to grasp skin between brain and base of skull to minimize damaging the brain. Peel away skin and skull to expose brain, rotating head as needed to remove skin and skull (Figure 1B).
8. When skin and skull are removed from the top and sides of the brain (Figure 1C), slide forceps underneath the brain (along the base of the skull) and ‘pinch’ off brain to remove it from head, then transfer brain to a new Petri dish with carbogenated ACSF on ice.

**CRITICAL:** Repeat steps 6–8 for all embryos before moving on to the next steps to ensure that brains are incubated in ACSF as fast as possible.

### Microdissection of embryonic forebrain regions

**Timing: 1–2 h depending on litter size, number of regions being dissected, etc.**

Specific forebrain regions are dissected out from the embryonic mouse brain. Here we describe how to harvest the MGE, LGE, CGE and cortex from an individual brain. Researchers can harvest the specific brain regions based on their experimental plans. All dissections performed in ice-cold carbogenated ACSF.

9. Transfer 1 brain to a new Petri dish with carbogenated ACSF on ice.
10. To harvest specific forebrain regions, remove any midbrain and hindbrain by pinching off any tissue caudal to the cerebral hemispheres (Figure 1D).

***Note:*** If uncertain about embryonic neuroanatomy, please consult a reference atlas^15^ or online resources such as the Allen Brain Developing Mouse Brain Atlas.

11. Using forceps, pinch between the hemispheres to hemisect the forebrain.
12. Position one hemisphere medial side up and remove any diencephalon tissue medial to the lateral ventricle. The ganglionic eminences (GEs) should be visible through the lateral ventricle (Figure 1E).
13. Gently pinch the dorsal cortex with forceps at 2–3 locations and peel it backwards to further expose the GEs (Figure 1F).
14. To remove the MGE, place one prong of the forceps into the posterior portion of the MGE-LGE sulcus and the other prong ventro-caudal to the MGE. Pinch off the MGE to separate it from the CGE (Figure 1G).
15. Continue separating the MGE from the LGE and septum by gently pinching around the MGE. When fully separated (Figure 1H), collect the MGE with either forceps or a pipette and transfer it to a ‘MGE’-labeled 5 mL round bottom tube with carbogenated ACSF on ice.

***Note:*** After removing MGE, the remaining LGE-CGE structure is symmetric and one can lose track of the orientation. Keep in mind which side of the brain is anterior (LGE) and posterior (CGE).

16. To cleanly separate LGE from CGE, make two incisions through the LGE-CGE structure to roughly split the structure into thirds (Figure 1I), with the anterior third being the LGE, the posterior third being the CGE, and the middle third being the LGE-CGE boundary region.
17. Using curved micro forceps, place the prongs dorsal and ventral to the LGE, then pinch off the LGE and transfer to a ‘LGE’-labeled 5 mL round bottom tube with carbogenated ACSF on ice (Figure 1J).
18. Following the same process, remove the CGE and transfer to a ‘CGE-labeled 5-mL round bottom tube with carbogenated ACSF on ice.

**CRITICAL:** Refrain from placing the forceps too deep when harvesting the LGE and CGE, as this will increase the likelihood of removing lateral cortex along with the LGE and/or CGE and contaminating the sample with glutamatergic cortical cells.

19. Remove a chunk of the cortex (e.g., somatosensory) and transfer to a ‘Cortex’-labeled 5 mL round bottom tube with carbogenated ACSF on ice (Figure 1J).
20. Repeat steps 12–19 for the other hemisphere, noting that the anterior and posterior positions are flipped.
21. Repeat steps 9–20 for all embryonic mouse brains.

***Optional:*** If harvesting nuclei, collected tissue can be transferred directly to a 1.5 mL conical tube, flash frozen on dry ice, and stored at −80°C. This allows for collection and storage of tissue from multiple animals for future processing. We do not recommend freezing tissue when collecting cells as cell membranes will fracture without using cryopreservation media.

***Note:*** If generating single-cell suspensions, prepare the Pronase Solution and Cell Reconstitution Solution at this point, after dissecting all embryonic brains.

### Adult mouse brain preparation – Brain sections

**Timing: ∼20–30 min/brain for brain matrix, ∼30–45 min/brain for vibratome**

Harvesting specific regions from the adult mouse brain by generating brain slices.

22. P21 and older mice are deeply anesthetized with Euthasol (270 mg/kg, 50 μL/30 g mouse, intraperitoneal (IP) injection). After confirming no response to painful toe pinch stimuli, decapitate mouse.
23. Peel skin forward and use forceps to pinch and remove skull starting from posterior end. When brain is fully exposed, remove and place into Petri dish with carbogenated NMDG High Mg^2+^ Solution on ice.
24. To generate brain sections using brain matrix:
  a. Place brain in a pre-chilled stainless steel 0.5 mm mouse brain matrix on ice (or similar matrix based on desired orientation and tissue thickness).
  b. Firmly depress razor blades through all slots encompassing desired brain regions (Figure 2A).

**Figure 2:**
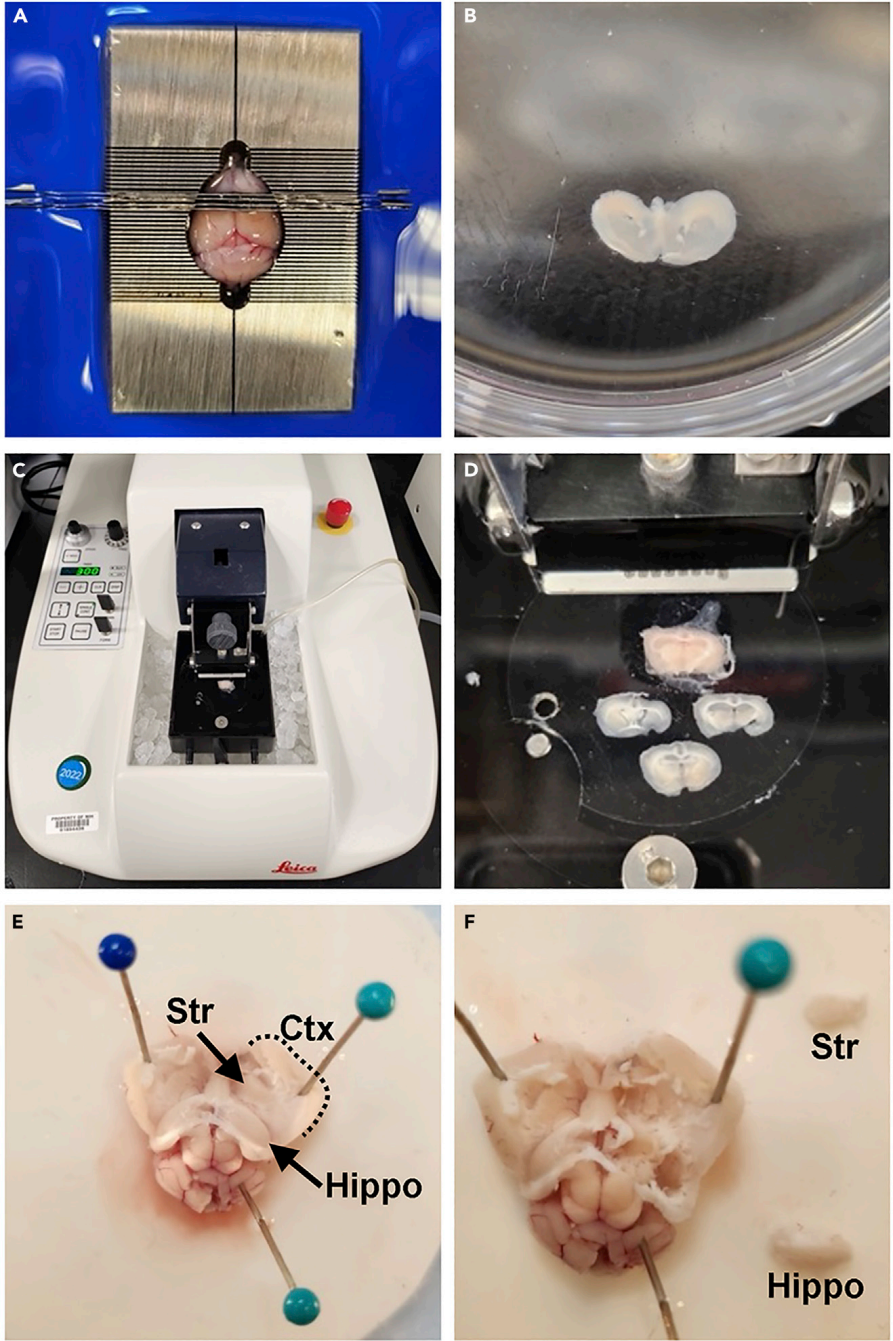
Adult mouse brain dissection (A and B) Adult mouse brain in matrix with razor blades for coronal brain slices. (C and D) Vibratome setup to generate coronal brain slices. (E and F) Whole mount preparation to remove intact brain regions (e.g., hippocampus (Hippo), striatum (Str) and cortex (Ctx)).
  c. Carefully remove razorblades, noting that tissue often sticks to one of the blades. Transfer desired slices to a Petri dish with carbogenated NMDG Activity-blocking Solution on ice (Figure 2B).
25. To generate brain sections using vibratome:
  a. If desired, remove excess tissue from anterior or posterior brain to minimize cutting through unneeded tissue.
  b. Glue brain(s) to vibratome plate and allow glue to dry for 30–60 s. Then transfer plate into the vibratome chamber surrounded by ice and filled with NMDG High Mg^2+^ Solution that can be continuously carbogenated (Figure 2C).
  c. Cut 300–400 μm sections and transfer desired sections to a Petri dish with carbogenated NMDG Activity-blocking Solution (Figure 2D).
26. Use forceps to remove desired regions (striatum, cortex, hippocampus, etc.) from individual brain slices and transfer to properly labeled 5 mL round bottom tube or Petri dish.

***Optional:*** If using a transgenic fluorescent reporter mouse line, dissecting desired regions can be done under a fluorescent dissecting scope to harvest fluorescent brain region/cells of interest.

***Optional:*** If harvesting nuclei, tissue can be directly added to a 1.5 mL conical tube, flash frozen on dry ice, and stored at −80°C. This allows for collection and storage of tissue from multiple animals for future processing. We do not recommend freezing tissue when collecting cells as cell membranes will fracture without using cryopreservation media.

### Adult mouse brain preparation – Whole mount

**Timing: ∼10 min/brain, depending on number of brain regions being collected**

Harvesting specific regions from the adult mouse brain by removing whole intact structures.

27. Follow steps 22 and 23 above to remove brains from juvenile/adult mice.
28. Place brain ventral side down in a Sylgard-coated petri dish containing NMDG High Mg^2+^ Solution and insert pins through cerebellum and anterior forebrain.
29. Use curved forceps to peel cortex antero-lateral and lay flat onto petri dish to expose underlying hippocampus and ventral structures (Figure 2E).
30. If collecting cortex and/or hippocampus, remove these structure and place into petri dish containing NMDG Activity-blocking Solution on ice (Figure 2F).
31. For striatum and other ventral brain structures, scrape away unwanted tissue, remove desired structure and transfer to petri dish containing NMDG Activity-blocking Solution on ice (Figure 2F).

***Optional:*** If using a transgenic fluorescent reporter mouse line, this dissection can be done under a fluorescent dissecting scope to harvest fluorescent brain region/cells of interest.

***Optional:*** If harvesting nuclei, tissue can be directly added to a 1.5 mL conical tube, flash frozen on dry ice, and stored at −80°C. This allows for collection and storage of tissue from multiple animals for future processing. We do not recommend freezing tissue when collecting cells as cell membranes will fracture without using cryopreservation media.

### Generation of single-cell suspensions from embryonic brain tissue

**Timing: ∼45 min, depending on number of samples**

Generation of single-cell suspensions from collected embryonic mouse brain regions.

**Figure 3:**
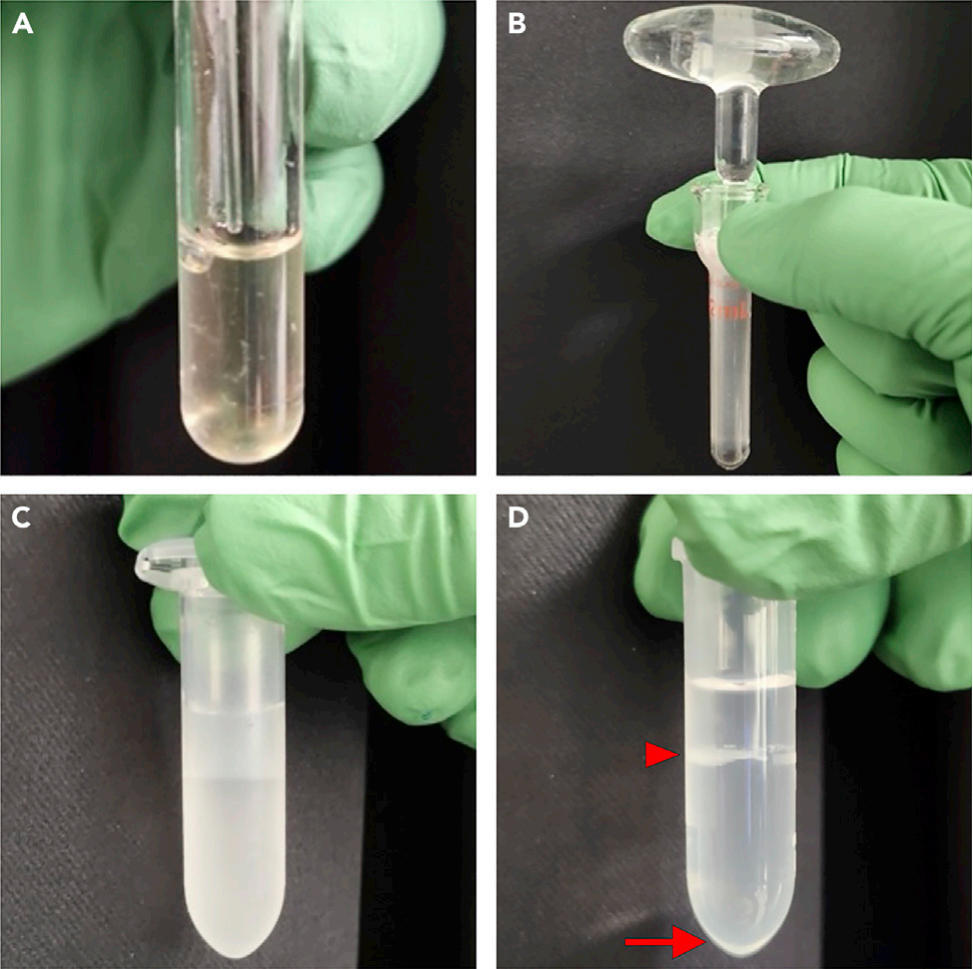
Preparing single-cell or nuclei solutions (A) Trituration of tissue with Pasteur pipette to generate single-cell suspensions. (B) Dounce homogenizer used to generate single-nuclei suspensions. (C and D) Cleanup of adult mouse tissue for single-nuclei suspension using Nuclei Pure Prep Isolation Kit, before (C) and after (D) centrifugation. Note the debris layer at the interface (arrowhead) while the nuclei precipitate at the bottom (arrow) after centrifugation.

32. After collecting all tissue, remove ACSF from the 5 mL tube and replace with 1 mL of Pronase solution. Incubate samples at 20°C–22°C for 15 min, gently flicking the tube 1–2 times during incubation to mix the tissue.
33. Carefully remove the Pronase solution without touching the intact tissue samples at the bottom of the tube and add 1–2 mL of Cell Reconstitution solution to each tube.
34. Mechanically dissociate tissue by carefully triturating samples with fire-polished glass pipettes, starting with 10–20 triturations with a large bore opening followed by 10–20 triturations with a smaller bore opening (Figure 3A). Repeat trituration for all samples, using different pipettes for each sample.

**CRITICAL:** Do not introduce bubbles while triturating as this will damage cell membranes, leading to increased cell death and debris in the suspension.

***Optional:*** If performing flow cytometry to harvest fluorescent cells, add DAPI (1 μL) and DRAQ5 (5 μM) per mL prior to straining cells. Then process cells on a flow cytometer and collect the DRA5+/DAPI- live cell population.

35. Use Pasteur pipette to transfer cell suspension through a pre-wetted Falcon 5 mL Tube w/ 35 μm Cell Strainer to remove debris, cell clumps, etc.
36. If not sorting cell suspension, transfer cells to a 2.0 mL low-bind tube and centrifuge at 500 g for 5 min at 4°C.
37. Remove supernatant. Cells can be reconstituted at desired volume in Cell Reconstitution solution depending on downstream application and counted on a hemocytometer or an automated cell counting machine.

### Generation of single-cell suspensions from adult brain tissue

**Timing: ∼90 min**

Generation of single-cell suspensions from adult mouse brain regions.

38. Cell dissociations are performed with Worthington’s Papain Dissociation System following manufacturer’s instructions with minor modifications described below.

**CRITICAL:** Maximum size of tissue for 1 reaction is 100 mg. We observed significant decrease in efficiency with tissue weighing more than 100 mg. If tissue weighs more than 100 mg, split tissue into multiple pieces and process in separate reactions, combining samples at the end.

39. Supplement the Papain dissociation solution with 1 μM TTX, 100 μM APV and 20 μM Actinomycin-D to minimize cell activity during tissue incubation.
40. Incubate tissue for 60 min in the Papain solution at 37°C with constant agitation.
41. Triturate tissue with fire-polished large and small bore Pasteur pipettes as described in step 34 above.

**CRITICAL:** Do not introduce bubbles while triturating as this will damage cell membranes, leading to increased cell death and debris in the suspension.

42. Centrifuge cells at 500 g for 5 min and discard supernatant.
43. Perform the density gradient as described in manufacturer’s instructions, then remove the supernatant and debris, with the dissociated cells pelleted at the bottom of the tube.
44. Reconstitute cells in desired volume of NMDG Activity-blocking Solution.
45. Due to increased debris in adult brains (e.g., myelin and severed neurites), we *strongly recommend* sorting the suspension to obtain a clean single-nuclei suspension.
  a. Resuspend cell pellet in a 50:50 mix of carbogenated Earle’s Balanced Salt Solutions (EBSS, from Papain kit):NMDG Activity-blocking Solution supplemented with 10% Fetal Bovine Serum (FBS), 10 U/mL DNase, DAPI (1 μL) and DRAQ5 (5 μL).
  b. Pass suspension through a pre-wetted 35 μm filter on a 5 mL round bottom tube and proceed with FACS, collecting DRAQ5+/DAPI- live cells.

### Generation of single-nuclei suspensions from embryonic and adult brain tissue

**Timing: ∼30–45 min, depending on number of samples**

Generation of single-nuclei suspensions from embryonic and adult brain tissue.

**CRITICAL:** For analysis of mRNA-only (e.g., RNA-seq), use the ‘RNA-only’ solutions. For analysis of DNA-only (e.g., ATAC-seq, ChIP-seq, CUT&Tag, etc.), use the ‘DNA-only’ solutions. For analysis of mRNA and DNA (e.g., 10x Genomics Multiome), use the ‘Multiome’ solutions.

**CRITICAL:** If analysis includes chromatin accessibility (e.g., ATAC-seq or Multiome), total time from Douncing samples (step 46) to final nuclei dilution (step 63) should take no more than 60 min. Sample preparation intervals greater that 60 min produce a noticeable decrease in library quality.

46. Add 1 mL of Nuclei Lysis Buffer (RNA-only, DNA-only or Multiome depending on application) to a pre-chilled Dounce homogenizer on ice, 1 Dounce per tissue region/sample.
  a. If using fresh tissue harvested as described above, transfer tissue pieces to Dounce.
  b. If using frozen tissue stored at −80°C, thaw for 30–60 s at 20°C–22°C and transfer tissue to Dounce.

**CRITICAL:** Maximum size of tissue for 1 reaction is 100 mg. We observed significant decrease in efficiency with tissue weighing more than 100 mg. If tissue weighs more than 100 mg, split tissue into multiple Dounces and run as separate reactions, combining sample replicates at the end.

47. Slowly dounce ∼10 times with A pestle and another ∼10 times with B pestle (1–2 s per up- or down-stroke), trying to minimize introduction of bubbles into the suspension (Figure 3B).
48. Place a 40 μm filter into a pre-chilled 50 mL Falcon tube on ice and wet with 1 mL Nuclei Wash (RNA-only, DNA-only or Multiome depending on application).
49. Transfer entire 1 mL lysed nuclei suspension through the pre-wetted filter.
50. Rinse the Dounce tube with 1 mL Nuclei Wash Buffer (RNA-only, DNA-only or Multiome depending on application). Transfer rinse solution through the pre-wetted filter.
51. Divide the nuclei suspension evenly (∼1.5 mL) between 2 pre-chilled 2 mL LoBind tubes.
52. Spin the tubes at 500 g for 5 min 4°C and remove supernatant.
53. Add 1 mL Nuclei Wash Buffer (RNA-only, DNA-only or Multiome depending on application) to each tube and gently triturate 3–5 times to dissociate pellet.
54. Repeat steps 52 and 53 for a total of 2 nuclei washes.
55. Spin the tubes at 500 g for 5 min 4°C and remove supernatant.
56. Add Nuclei Resuspension Buffer.
  a. For single-nuclei 10x Genomics RNA-seq experiments: Add 250 μL of RNA-only Nuclei Resuspension Buffer (if analyzing mRNA only) or DNA-only Nuclei Wash Buffer (if analyzing DNA) or Multiome Nuclei Wash Buffer (if analyzing mRNA + DNA) to each tube, triturate and combine replicates into a single micro tube making the final volume 500 μL.
  b. For low-input or bulk chromatin assays (e.g., CUT&Tag, ChIP-seq, etc.), add appropriate buffer and continue with assay-specific protocols.

***Note:*** Depending on tissue quantify and pellet size, it might be preferrable to use less than 250 μL Nuclei Resuspension or Nuclei Wash buffer per tube to resuspend the pellet after the last spin.

57. To remove debris for downstream applications, we *strongly recommend* sorting the nuclei suspensions.
  a. Add 1 μL DAPI (or DRAQ5) to nuclei suspension and filter suspension through a pre-wetted 35 μm filter. Proceed to cell sorter, collecting DAPI+ nuclei (or fluorescent nuclei if using a reporter mouse line).

***Note:*** If performing 10x Genomics ATAC-seq or Multiome reactions, we recommend collecting > 50,000 nuclei if possible, as this will provide sufficient nuclei for these reactions after loss of nuclei from spinning and reconstitution.

***Optional:*** Some samples from adult tissue with high myelin content (e.g., adult spinal cord) contain a significant amount of debris and harvesting nuclei via FACS was extremely inefficient. In these instances, we recommend performing the following optional additional cleanup procedure using the Nuclei Pure Prep Isolation Kit prior to FACS to remove excess myelin. Steps 58–61 are optional.

**CRITICAL:** Sucrose Gradient clean-up has only been tested for analyzing mRNA alone. It has NOT been validated for analyzing DNA alone or Multiome experiments and is not expected to work well in such cases. If debris or myelin removal is needed for DNA only or Multiome (mRNA + DNA) samples, we *strongly recommend* purifying the nuclei suspensions via flow cytometry.

58. Add 900 μL of 1.8 M Nuclei PURE Sucrose Cushion Solution to 2 mL LoBind tube and gently load nuclei suspension on top of Sucrose Cushion Solution (Figure 3C).

***Note:*** Nuclei from different cell types may have different densities. Thus, different sucrose concentrations may be used by diluting 2 M Nuclei PURE Sucrose Cushion Solution with Nuclei PURE Sucrose Cushion Buffer.

**CRITICAL:** Do not mix nuclei suspension and Sucrose Cushion Solution, as this will lead to suboptimal cleanup and loss of nuclei.

59. Centrifuge the sucrose gradient at 13,000 g for 45 min at 4°C (Figure 3D).
60. Carefully remove supernatant leaving ∼50 μL of bottom layer in each tube and add 450 μL RNA-only Nuclei Resuspension Buffer (if analyzing mRNA only) or DNA Nuclei Wash Buffer (if analyzing DNA) and triturate to resuspend the pellet.
61. Add DAPI (or DRAQ5) to the nuclei suspension and triturate, then pass suspension through a pre-wetted 35 μm filter on a 5 mL round bottom tube and proceed to flow cytometry.
  a. For low-input or bulk chromatin assays (e.g., CUT&Tag, ChIP-seq, etc.), collect nuclei in appropriate buffer and continue with assay-specific protocols.

***Note:*** If performing 10x Genomics ATAC-seq or Multiome reactions, we recommend collecting > 50,000 nuclei if possible, as this will provide sufficient nuclei for these reactions after loss of nuclei from spinning and reconstitution.

### Preparation of samples for single-cell/nuclei sequencing with 10x genomics kits

**Timing: ∼10–20 min depending on number of samples**

Based on 10x Genomics recommendations and our own experience, we aim for ∼15,000 cells/nuclei per 10× reaction to minimize doublets and maximize recovery, which typically results in 5,000–8,000 good quality cells/nuclei per reaction. The current 10× RNA-seq protocol allows for cells in 43.2 μL whereas the ATAC-seq and Multiome reactions require nuclei in 5 μL, so we describe our procedure for both types of sequencing reactions. Please consult the 10x Genomics protocol for your specific assay for more details.

62. For 10x Genomics RNA-seq experiments:
  a. With FACS:
    i. Collect ∼15,000 cells or nuclei in a 1.5 mL LoBind collection tube containing 10 μL RNA-only Nuclei Resuspension Buffer and use 43.2 μL of this cell/nuclei suspension to proceed with the 10x Genomics RNA-seq reaction based on manufacturer’s instructions.
      ***Note:*** This process results in a final volume of ∼45 μL with the Sony SH800 cell sorter (volume may vary with other sorters), thus nearly all this cell/nuclei suspension can be applied directly to the 10x Genomics RNA-seq kit with no need to spin the cells/nuclei suspension, thus eliminating excess loss of cells/nuclei after sorting.
  b. Without FACS:
    i. Count the number of cells/nuclei on a hemocytometer or automated cell sorter to determine the concentration.
    ii. Prepare cell/nuclei suspension at a concentration of 1,000 cells/nuclei per μL and use 15 μL of this cell/nuclei suspension to proceed with the 10x Genomics RNA-seq reaction based on manufacturer’s instructions.
63. For 10x Genomics ATAC-seq or Multiome sequencing experiments:
  a. For both sorted and non-sorted nuclei, centrifuge nuclei suspensions at 500 g for 10 min at 4°C in a swinging-bucket rotor (since high nuclei densities and small solution volumes are desired, we recommend a swinging-bucker rotor at this step to ensure nuclei pellet collects at the bottom of the tube).
  b. Carefully remove the supernatant, leaving < 20 μL in the tube. Resuspend nuclei in this leftover solution
  c. Count the number of nuclei on a hemocytometer or automated cell sorter to determine the concentration. (e.g., we use 2 μL cell suspension + 8 μL PBS + 10 μL Trypan Blue solution, then adjust calculations accordingly).
  d. Dilute sample to a concentration of 3,000 nuclei/μL using DNA Diluted Nuclei Buffer (ATAC-seq) or Multiome Diluted Nuclei Buffer (Multiome) and use 5 μL to proceed with the corresponding 10x Genomics ATAC-seq or Multiome reaction based on manufacturer’s instructions.

## Expected outcomes

**Figure 4:**
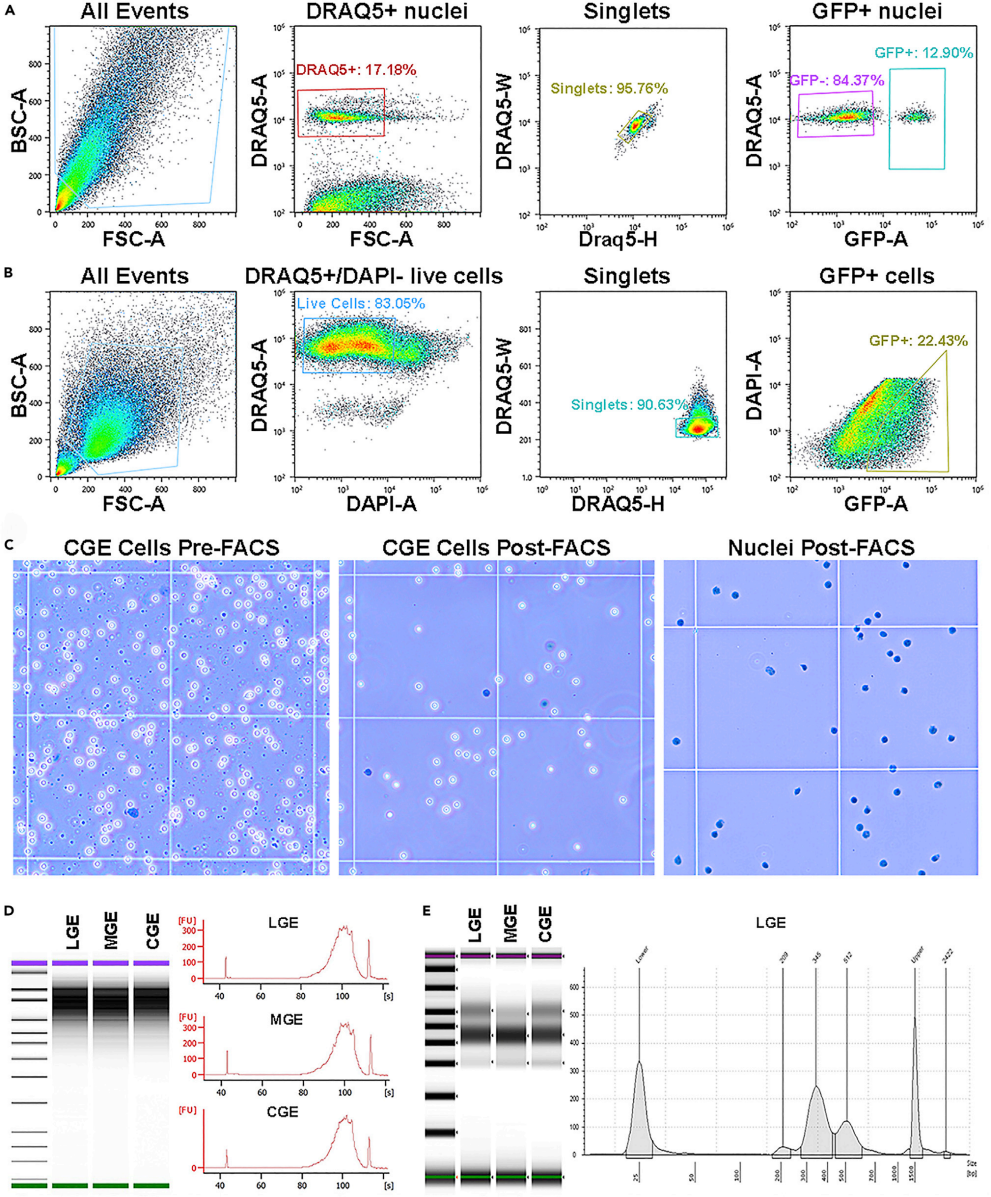
Expected outcomes for successful single-cell/nuclei suspensions from brain tissue (A) FACS gating strategy from adult cortex of *Dlxa6Cre;Sun1GFP* mouse to harvest cortical interneuron nuclei (GFP+). (B) FACS gating strategy from embryonic LGE of *Nestin-GFP* mouse to harvest Nestin-expressing LGE cells (GFP+). Note the significant amount of debris in the adult sample compared to the embryonic tissue, due in large part to excess myelin and tissue shearing in adult brains. (C) Example of cell dissociation from CGE before (*left*) and after (*middle*) FACS, with dead cells labeled by Trypan Blue (blue). *Right*, example of nuclei dissociation after FACS. (D) Example of high-quality cDNA libraries from single-cell embryonic dissociations prepared with 10x Genomics RNA-seq kit, run on Agilent Bioanalyzer. (E) Example of high-quality cDNA libraries from single-nuclei embryonic dissociations prepared with 10x Genomics ATAC-seq kit, run on Agilent Tapestation.

Researchers should observe very clean single-cell or single-nuclei suspensions, with minimal debris and dead cells. Cleaning up both cell and nuclei suspensions via FACS will remove significant amounts of debris and improve the purity of the samples (Figures 4A and 4B), which can be visualized on a hemocytometer or automated cell counter (Figure 4C). The total number of cells/nuclei will depend on the amount of starting material and efficiency of the cleanup procedure. Generation of cDNA libraries for downstream applications should yield clean chromatograms of ample cDNA for sequencing (Figures 4D and 4E).

## Limitations

Some neuronal subtypes appear to be more sensitive to these dissociation processes than others, which can introduce bias into the number and/or ratio of cell types recovered. Also, smaller cell bodies such as glia may be more likely to survive the dissociation process and be overrepresented in the cell population, whereas neuronal cell types with large cell bodies (e.g., Purkinje cells and motor neurons) may be less likely to survive. Researchers should keep in mind that the proportion of cell types recovered with these dissociation protocols may not be fully representative of the actual proportions in the brain region of interest.

Additionally, some cell types represent only a small fraction of total neurons in a particular region and thus harvesting ample numbers of these cells/nuclei without fluorescent reporters can be challenging. For example, single-nuclei RNA-seq experiments on the spinal cord recovered very few cholinergic motor neurons due to their relative sparsity,^16^ but using fluorescent reporters allowed for the enrichment of motor neurons.^9^^,^^17^ Whenever possible, using fluorescent reporters is highly recommended to enrich target cells, with the Cre-dependent *Sun1-sfGFP* ‘INTACT’ mouse being one excellent tool to fluorescently label nuclei.^18^

## Troubleshooting

### Problem 1

Dirty sample contaminated with debris from adult brain dissociations (steps #38–45 and #58–61).

### Potential solution

Samples harvested from adult brains contain significant amounts of myelin and cellular debris that can be challenging to remove. This is especially for regions with a high ratio of axons to cell bodies such as the striatum and spinal cord. Our main recommendation is to pass these single-cell or -nuclei suspensions through a cell sorter to remove nearly all debris, using DRAQ5+/DAPI- to collect live cells or just DAPI+ to collect all nuclei. Another critical step is to ensure that the layer of debris from the Papain dissociation for adult brains (step #43) is completely removed; if not, this will increase the amount of debris in downstream applications. Additionally, one could reduce the amount of starting tissue, or split the tissue samples into multiple tubes and increase the number of cleanup reactions. Another alternative is to utilize specific reagents to remove myelin, such as Myelin Removal Beads (Miltenyi Biotec), but this adds additional time to the procedure that could result in further cell/nuclei loss.

### Problem 2

Excessive amounts of cell/nuclei clumps, inefficient single-cell/nuclei dissociation (steps #32–61).

### Potential solution

Inefficient cell or nuclei dissociation can result in clumps of cells or nuclei sticking together, leading to cell/nuclei loss during filtration and/or cell sorting steps. Sometimes clumps may be visible during cell counting. Other times cell/nuclei clumps will not pass through the filters and thus would not be visibly detected, but instead would result in lower than expected yields. To minimize clumping, investigators can extend the incubation time of pronase for embryonic tissue (step #32), papain for adult tissue (step #40), increase the number of triturations (steps #34 and 41) and increase the number of dounces with the homogenizer (step #47). Depending on the source of excess cell/nuclei clumping, any of these alterations should reduce the likelihood of clumping and increase single-cell/nuclei dissociation efficiency.

### Problem 3

Evidence of unhealthy, dying or dead cells on hemocytometer or automated cell counter after procedure (steps #37 and 45).

### Potential solution

Cells that have been damaged during the preparation can lead to poor results for downstream applications. If single-cell solutions contain a high amount (>10%) of dead or dying cells based on live/dead cell marker analysis during counting, there are several steps that one can take. First, ensure that cell triturations (steps #34 and 41) are done with fire-polished Pasteur pipettes and that no bubbles are introduced during trituration, as this can damage cells. Second, make sure cells are gently added to filters and solution allowed to pass through via gravity or gentle tapping of the tube (steps #35 and 45); do not use excessive force to pass cell solutions through filter as this can lead to shearing and damage cells. Third, for adult tissue (steps #38–45), brain regions can be cut up into smaller pieces to ensure efficient dissociation with Papain kit and minimize cell damage during this procedure.

### Problem 4

Lower than expected yield for single-nuclei suspensions (steps #46–63).

### Potential solution

This can be an issue when using fluorescent reporters to select specific neuronal subtypes in a particular brain region. For nuclei preparations, we recommend flash freezing desired brain regions on dry ice and then storing tissue at −80°C. This allows researchers to collect sufficient tissue samples and pool them together for processing, thus increasing the starting number of desired cells and ultimate yield. As noted in step #46, we recommend using < 100 mg of tissue for each cleanup reaction. If combination of frozen tissue samples exceeds this weight, split sample into 2 (or more) tubes for cleanup process.

### Problem 5

Lower than expected yield for single-nuclei/cell suspensions after flow cytometry (steps #34–35, 45, 57, 61).

### Potential solution

In our experience using the Sony SH800 Cell Sorter, the number of cells or nuclei obtained after sorting is ∼85%–100% of the expected number based the sorter information. If the output is lower than expected, researchers can increase the number of sorted cells collected (if possible) to increase total yield. We have found that additional spins and reconstitutions after sorting can lead to loss of 20%–40% of cells or nuclei, so we recommend collecting sorted cells in small volumes of desired solutions for downstream applications whenever possible to eliminate the need for additional spins and reconstitutions.

## Resource availability

### Lead contact

Further information and requests for resources and reagents should be directed to lead contact Timothy J. Petros (tim.petros@nih.gov).

### Materials availability

This study did not generate any novel reagents.

## Data Availability

This study did not generate any datasets or original code.
